# Supplementary material for: Accurate absolute free energies for ligand–protein binding based on non-equilibrium approaches
Source: Commun Chem. 2021 May 11;4:61. doi: 10.1038/s42004-021-00498-y (PMC9814727; doi:10.1038/s42004-021-00498-y)
Supplement: Supplementary file 1 — Supplementary Information [file 42004_2021_498_MOESM1_ESM.pdf]

Supporting Information: Accurate absolute free energies for ligand-protein binding based on non-equilibrium approaches

Vytautas Gapsys,[1,a)] Ahmet Yildirim,[2] Matteo Aldeghi,[1,3] Yuriy Khalak,[1] David van der Spoel,[4] and Bert L. de Groot[1,a)]

[1]*Computational Biomolecular Dynamics Group, Max Planck Institute for Biophysical Chemistry, 37077 Göttingen, Germany*

[2]*Department of Physics, Siirt University, 56100 Siirt, Turkey*

[3]*Present Address: Vector Institute for Artificial Intelligence, Toronto, Canada*

[4]*Uppsala Centre for Computational Chemistry, Science for Life Laboratory, Department of Cell and Molecular Biology, Uppsala University, Husargatan 3, Box 596, SE-75124 Uppsala, Sweden*

[a)] Corresponding authors: vgapsys@gwdg.de bgroot@gwdg.de

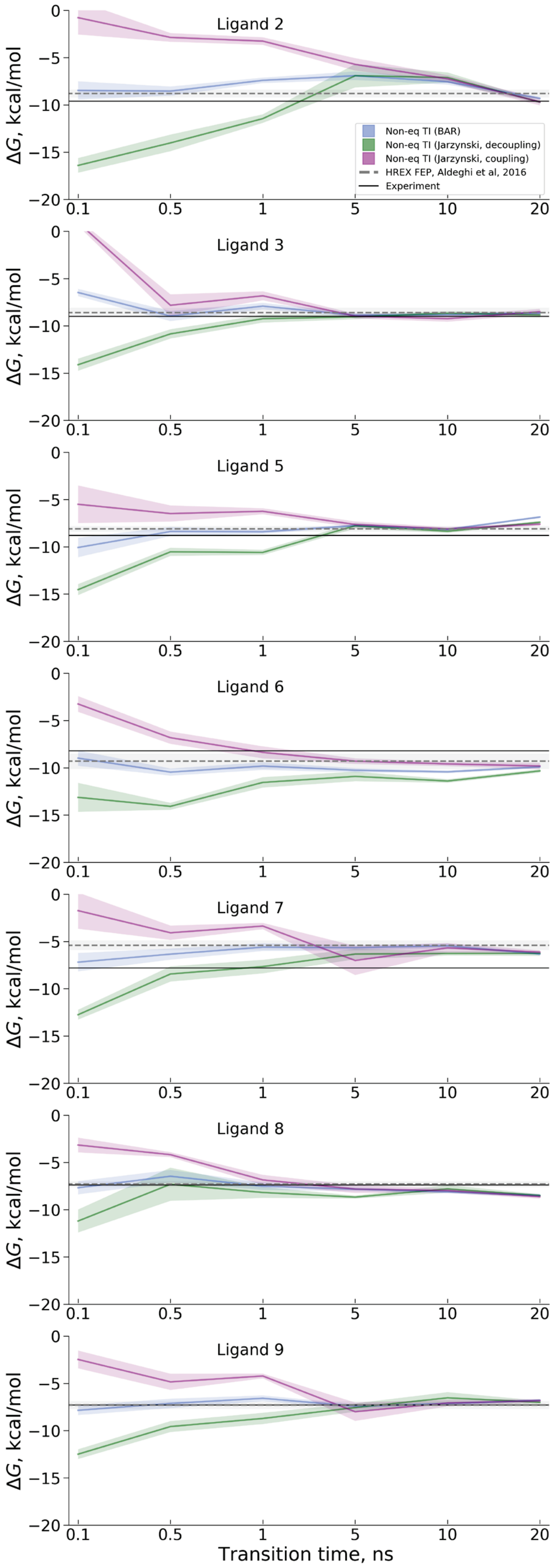

Fig S1. Absolute free energies calculated for the ligands in a 7 ligand subset binding to the BRD4(1) protein. The dependence of the non-equilibrium TI results on the transition time is depicted. The experimental (solid gray) and FEP HREX (dashed gray) lines are shown for reference and do not depict time dependence.

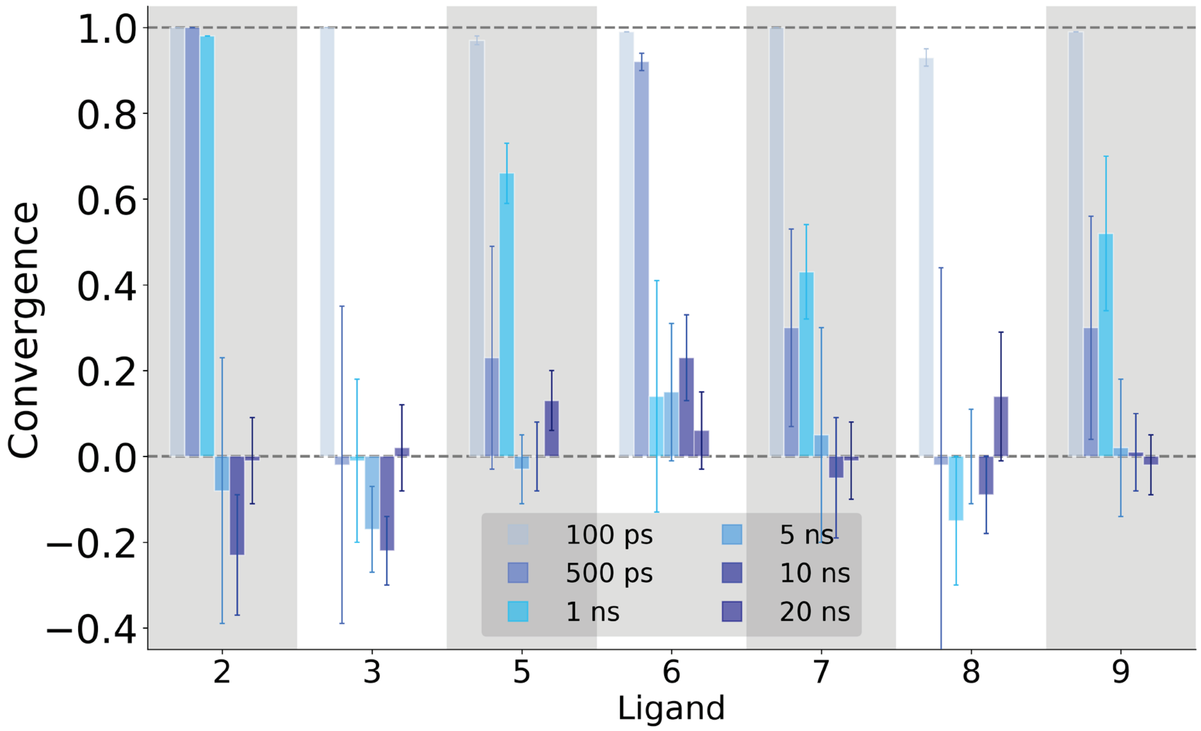

Fig S2. Convergence measure for the bi-directional (BAR) non-equilibrium TI based free energy estimates for the ligand coupling to protein branch of the thermodynamic cycle (7 ligands complexed with BRD4(1)). Six transition times were probed by performing 50 forward and backward transitions (the overall results for this test are summarized in the main text Figure 2). A convergence value of approximately 0 indicates a well-converged estimate, while values approaching 1, suggest that the convergence is not sufficient. Performing the transitions slower facilitates the convergence. Already at 500 ps transition time, the estimates are converged for most of the cases. For the Ligands 2 and 6 the convergence can be achieved by adding additional independent repeats and retaining the 500 ps transition time, as illustrated further in Figure S3.

Supplementary Note 1

The convergence estimator is based on the work by Hahn & Then [1]. It was observed that the overlap between the forward and reverse work distributions (U_α) can be defined in two different ways based on the Fermi functions for the forward $b_{\widehat{\Delta F}}$ and reverse $t_{\widehat{\Delta F}}$ process; here α denotes the ratio of forward transitions and total number of transitions (n_0/N) and $\widehat{\Delta F}$ is the free energy estimate. The overlap can be defined using the first moments $\widehat{U_\alpha} = \overline{t_{\widehat{\Delta F}}} = \overline{b_{\widehat{\Delta F}}}$ (overbars denote ensemble averages), as well as the second moments $\widehat{U_\alpha^{II}} = \frac{n_0}{N}\overline{t^2_{\widehat{\Delta F}}} + \frac{n_1}{N}\overline{b^2_{\widehat{\Delta F}}}$ (n_1 is the number of reverse transitions). Based on these overlap estimates a convergence measure is defined as $a = \frac{\widehat{U_\alpha} - \widehat{U_\alpha^{II}}}{\widehat{U_\alpha}}$. The measure is defined over the range $-1 < a < 1$. Since the overlap estimate based on the second moments converges slower than $\widehat{U_\alpha}$, for the unconverged $\widehat{\Delta F}$ estimates a has a value close to 1. With the increased number of observations, the overlap increases, thus facilitating the convergence and bringing the a value closer to 0. Upon reaching convergence, a fluctuates around 0. Thus, small positive and negative values indicate an already converged estimate.

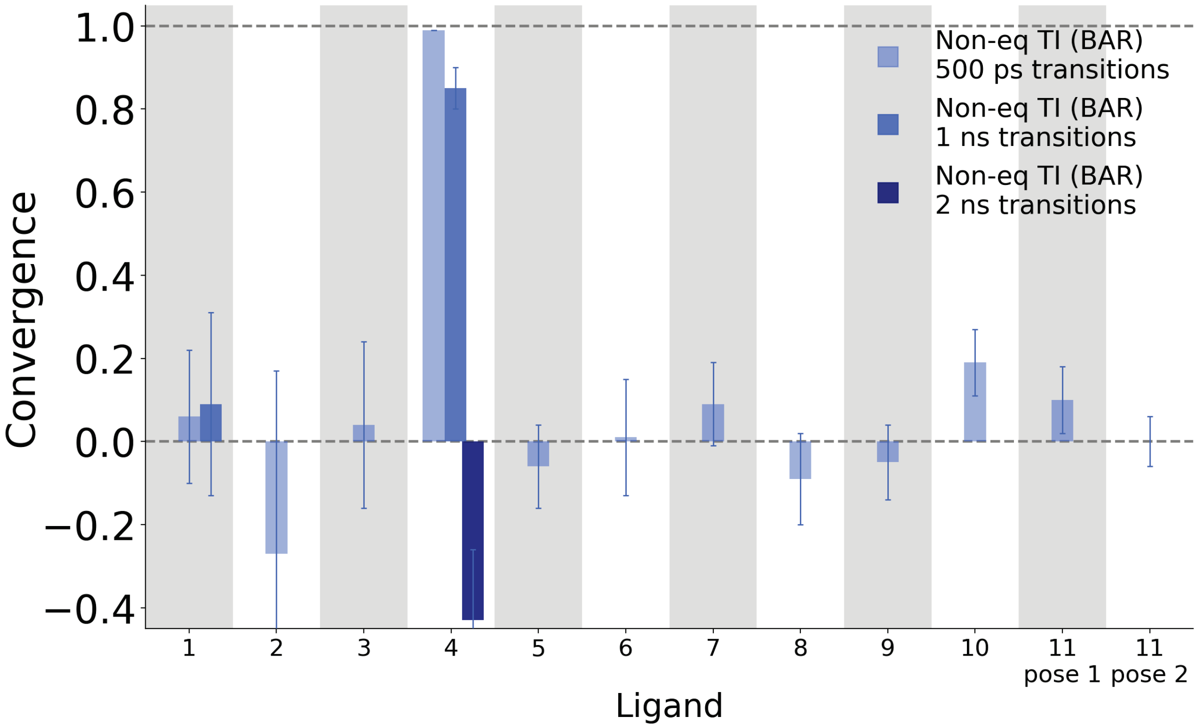

Fig S3. Convergence measure for the bi-directional (BAR) non-equilibrium TI based free energy estimates for the ligand coupling to protein branch of the thermodynamic cycle (11 ligands complexed with BRD4(1)). The value close to 0 indicates converged estimate, while a value close to 1, suggests that the convergence is not sufficient. Performing the transitions slower (dark blue bars) facilitate the convergence.

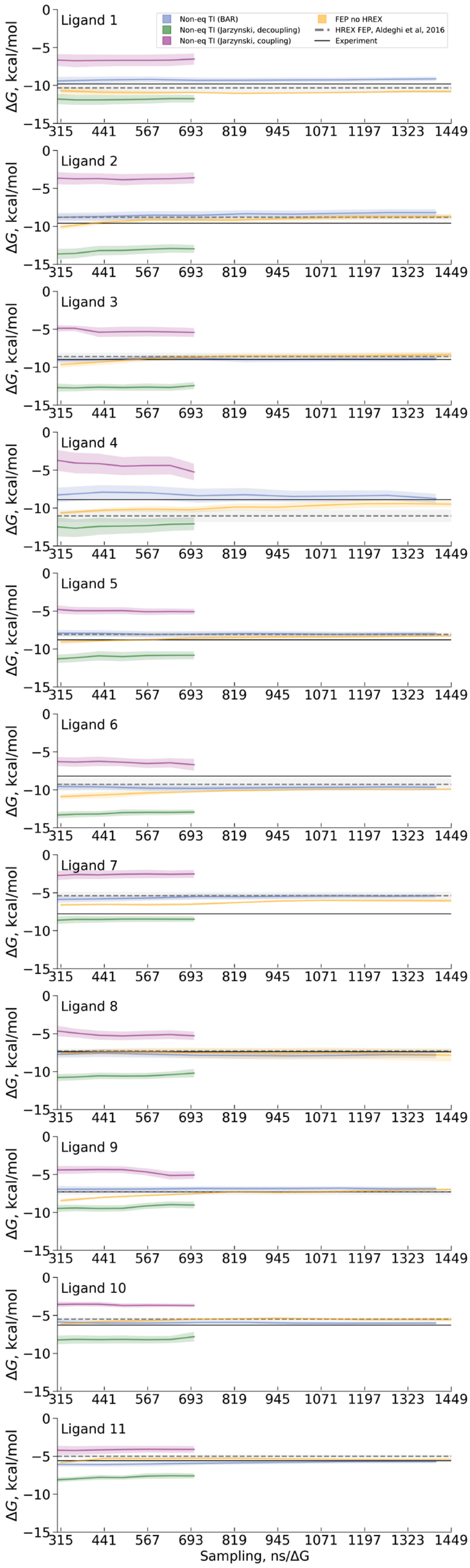

Fig S4. Absolute free energies for 11 ligands binding to the BRD4(1) protein depicted against the sampling time invested in calculation. The time reflects only the sampling invested in the protein-ligand coupling part. The solid and dashed gray lines for the experimental and FEP HREX values, respectively, are shown for reference and do not depict time dependence.

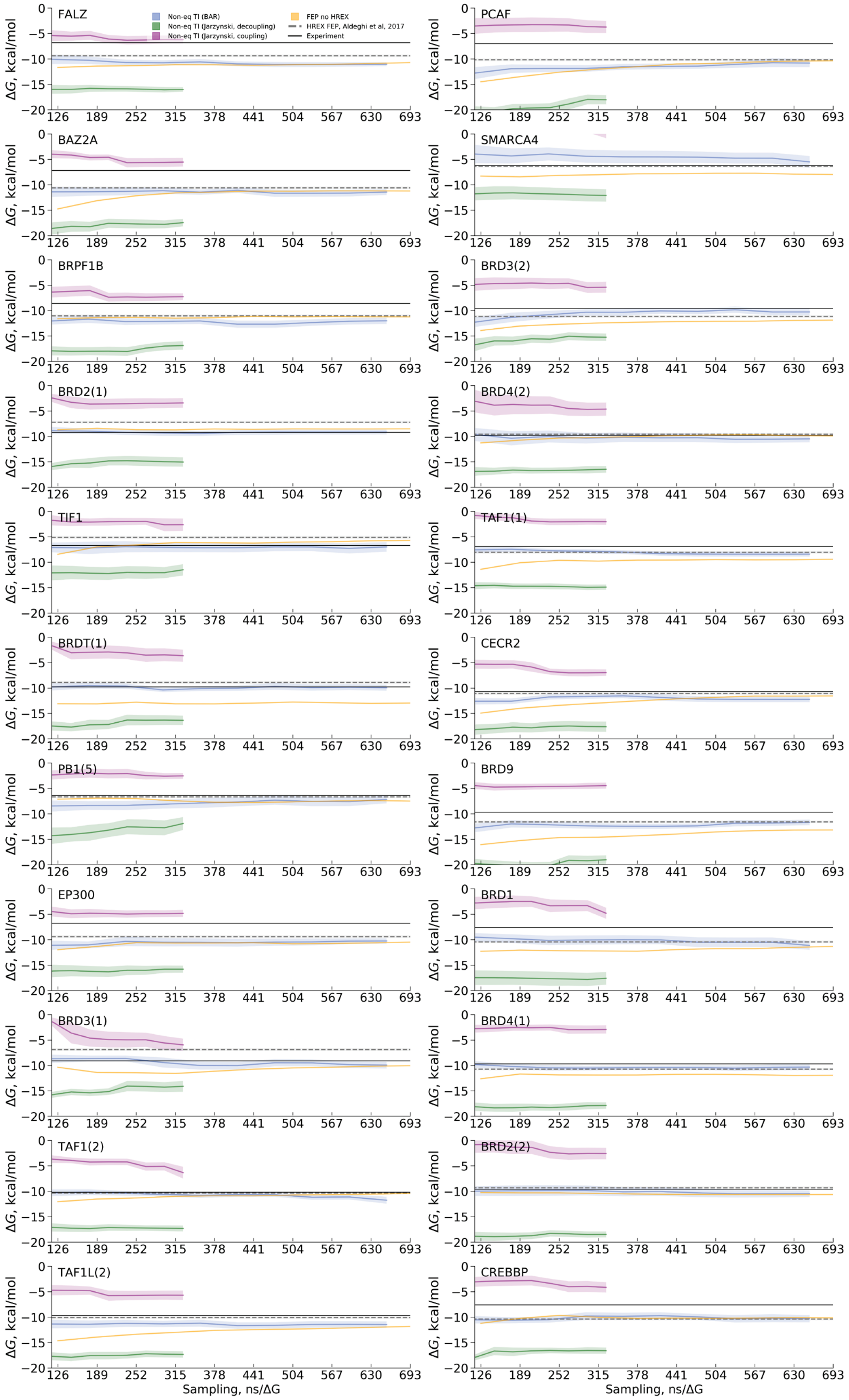

Fig S5. Absolute free energies for bromosporine binding to 22 bromodomain proteins depicted against the sampling time invested in calculation. The time reflects only the sampling invested in the protein-ligand coupling part. The solid and dashed gray lines for the experimental and FEP HREX values, respectively, are shown for reference and do not depict time dependence.

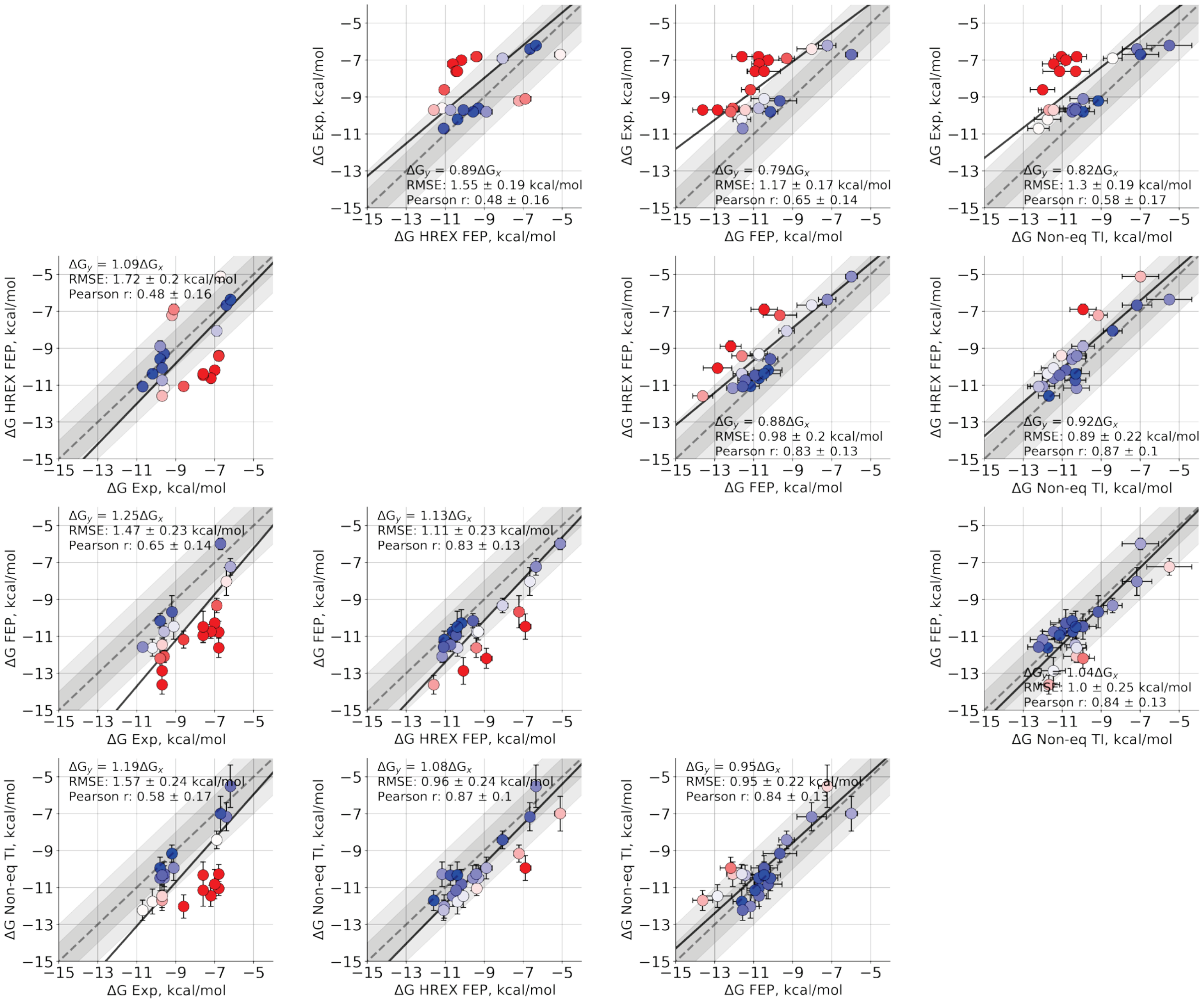

Fig S6. Comparison of the absolute free energies for 11 ligands binding to the BRD4(1) protein as obtained from different approaches. RMSE corresponds to the root mean squared error after a linear fit without an offset of the values depicted on x-axis to the values on y-axis.

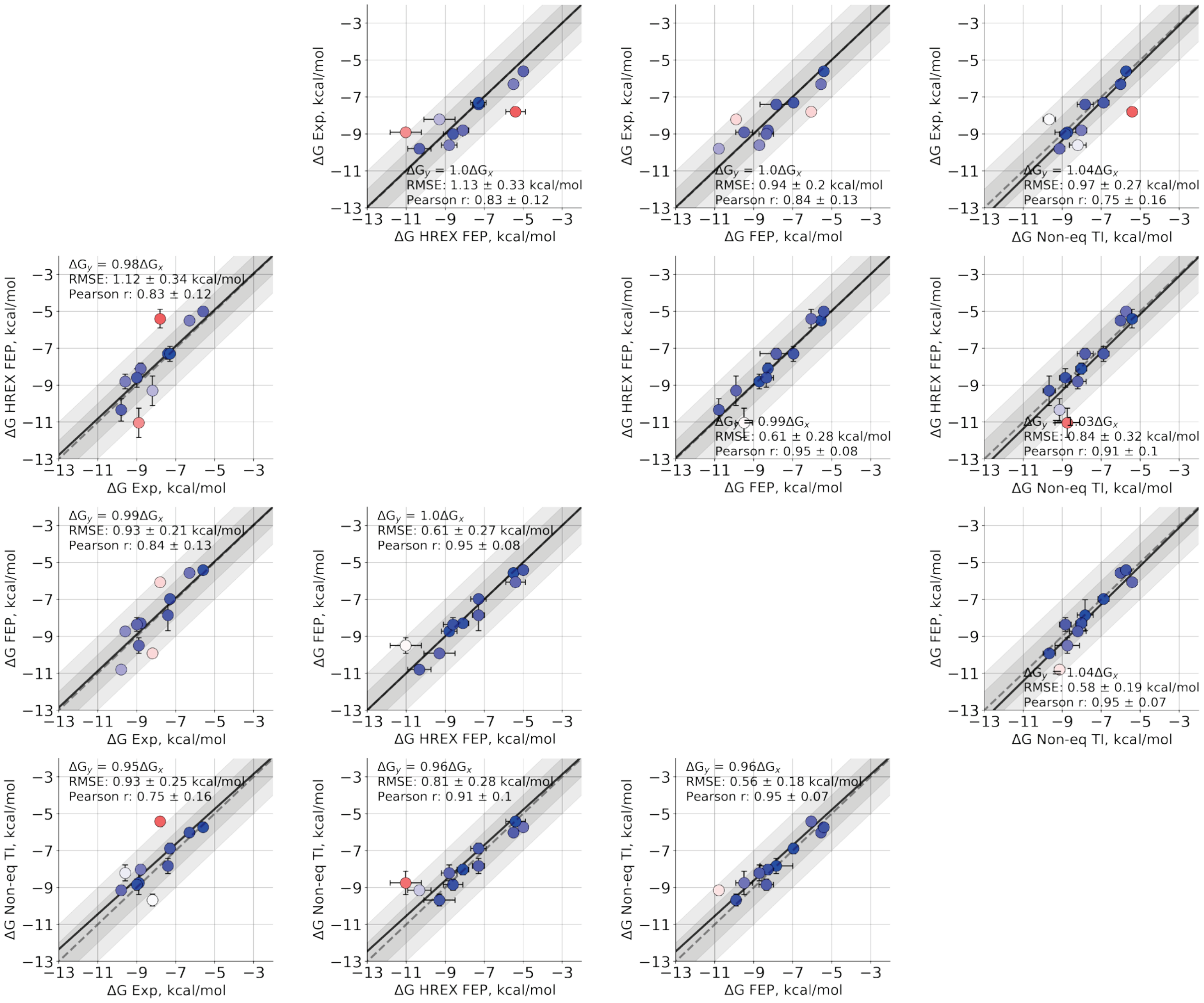

Fig S7. Comparison of the absolute free energies for bromosporine binding to 22 bromodomain proteins as obtained from different approaches. RMSE corresponds to the root mean squared error after a linear fit without an offset of the values depicted on x-axis to the values on y-axis.

Ligand	Experiment	FEP no HREX	HREX FEP	Non-eq TI Jarzynski decoupling	Non-eq TI Jarzynski coupling	Non-eq TI BAR
Ligand1	-9.80 ± 0.10	-10.80 ± 0.14	-10.33 ± 0.60	-11.75 ± 0.51	-6.53 ± 0.73	-9.14 ± 0.27
Ligand2	-9.60 ± 0.10	-8.72 ± 0.25	-8.80 ± 0.40	-12.98 ± 0.53	-3.62 ± 0.68	-8.21 ± 0.42
Ligand3	-9.00 ± 0.10	-8.36 ± 0.38	-8.60 ± 0.50	-12.43 ± 0.49	-5.43 ± 0.57	-8.84 ± 0.32
Ligand4	-8.90 ± 0.10	-9.49 ± 0.42	-11.03 ± 0.80	-12.08 ± 0.84	-5.25 ± 1.13	-8.75 ± 0.62
Ligand5	-8.80 ± 0.10	-8.28 ± 0.12	-8.10 ± 0.30	-10.84 ± 0.51	-5.10 ± 0.38	-8.03 ± 0.29
Ligand6	-8.20 ± 0.10	-9.92 ± 0.03	-9.30 ± 0.80	-12.94 ± 0.33	-6.70 ± 0.77	-9.67 ± 0.31
Ligand7	-7.80 ± 0.10	-6.06 ± 0.22	-5.40 ± 0.50	-8.49 ± 0.36	-2.53 ± 0.52	-5.42 ± 0.29
Ligand8	-7.40 ± 0.10	-7.85 ± 0.79	-7.30 ± 0.30	-10.21 ± 0.53	-5.29 ± 0.54	-7.83 ± 0.42
Ligand9	-7.30 ± 0.00	-6.97 ± 0.09	-7.30 ± 0.40	-9.04 ± 0.43	-5.08 ± 0.51	-6.89 ± 0.27
Ligand10	-6.30 ± 0.10	-5.56 ± 0.22	-5.50 ± 0.20	-7.82 ± 0.64	-3.71 ± 0.22	-6.02 ± 0.20
Ligand11	-5.60 ± 0.00	-5.41 ± 0.05	-5.00 ± 0.20	-7.60 ± 0.36	-4.10 ± 0.40	-5.72 ± 0.19

Table S1: Binding free energies for the BRD4(1) specificity study. Values are in kcal/mol.

Protein	Experiment	FEP no HREX	HREX FEP	Non-eq TI Jarzynski decoupling	Non-eq TI Jarzynski coupling	Non-eq TI BAR
FALZ	-6.80 ± 0.10	-10.73 ± 0.06	-9.39 ± 0.24	-16.01 ± 0.46	-6.22 ± 0.74	-11.05 ± 0.40
PCAF	-7.00 ± 0.10	-10.36 ± 0.06	-10.18 ± 0.26	-18.03 ± 0.86	-3.73 ± 1.19	-10.82 ± 0.78
BAZ2A	-7.20 ± 0.10	-11.23 ± 0.06	-10.62 ± 0.26	-17.46 ± 0.75	-5.55 ± 0.85	-11.45 ± 0.60
SMARCA4	-6.20 ± 0.10	-7.99 ± 0.06	-6.36 ± 0.26	-12.11 ± 1.20	0.96 ± 1.77	-5.51 ± 1.18
BRPF1B	-8.60 ± 0.10	-11.25 ± 0.06	-11.06 ± 0.25	-16.88 ± 0.80	-7.28 ± 0.68	-12.02 ± 0.62
BRD3(2)	-9.60 ± 0.10	-11.89 ± 0.06	-11.16 ± 0.25	-15.25 ± 0.73	-5.42 ± 1.07	-10.27 ± 0.65
BRD2(1)	-9.20 ± 0.10	-8.51 ± 0.06	-7.22 ± 0.26	-15.05 ± 0.94	-3.42 ± 0.93	-9.16 ± 0.46
BRD4(2)	-9.80 ± 0.10	-9.93 ± 0.06	-9.58 ± 0.26	-16.48 ± 0.65	-4.63 ± 1.20	-10.49 ± 0.68
TIF1	-6.70 ± 0.10	-5.71 ± 0.06	-5.11 ± 0.27	-11.50 ± 1.08	-2.63 ± 1.28	-7.00 ± 0.91
TAF1(1)	-6.90 ± 0.10	-9.44 ± 0.06	-8.06 ± 0.27	-14.91 ± 0.52	-2.04 ± 0.59	-8.42 ± 0.48
BRDT(1)	-9.80 ± 0.10	-12.96 ± 0.06	-8.89 ± 0.30	-16.36 ± 0.99	-3.65 ± 1.16	-9.94 ± 0.59
CECR2	-10.70 ± 0.10	-11.55 ± 0.06	-11.08 ± 0.26	-17.61 ± 0.98	-6.97 ± 0.64	-12.22 ± 0.58
PB1(5)	-6.40 ± 0.10	-7.48 ± 0.06	-6.66 ± 0.25	-11.91 ± 1.25	-2.54 ± 0.61	-7.17 ± 0.80
BRD9	-9.70 ± 0.10	-13.18 ± 0.06	-11.59 ± 0.25	-19.04 ± 0.87	-4.46 ± 0.59	-11.69 ± 0.53
EP300	-6.80 ± 0.10	-10.50 ± 0.06	-9.42 ± 0.27	-15.81 ± 0.68	-4.84 ± 0.66	-10.27 ± 0.53
BRD1	-7.60 ± 0.10	-11.34 ± 0.06	-10.45 ± 0.25	-17.61 ± 1.22	-4.81 ± 1.07	-11.14 ± 0.88
BRD3(1)	-9.10 ± 0.10	-10.06 ± 0.06	-6.89 ± 0.27	-14.11 ± 1.02	-5.94 ± 1.17	-9.94 ± 0.68
BRD4(1)	-9.70 ± 0.10	-11.93 ± 0.06	-10.74 ± 0.25	-17.88 ± 0.63	-2.92 ± 0.77	-10.33 ± 0.55
TAF1(2)	-10.20 ± 0.10	-10.44 ± 0.06	-10.37 ± 0.25	-17.31 ± 0.54	-6.33 ± 1.17	-11.76 ± 0.68
BRD2(2)	-9.60 ± 0.10	-10.65 ± 0.06	-9.31 ± 0.26	-18.49 ± 0.59	-2.58 ± 1.06	-10.47 ± 0.64
TAF1L(2)	-9.70 ± 0.10	-11.83 ± 0.06	-10.08 ± 0.24	-17.34 ± 0.64	-5.70 ± 0.90	-11.46 ± 0.61
CREBBP	-7.60 ± 0.10	-10.15 ± 0.06	-10.38 ± 0.24	-16.60 ± 0.60	-4.15 ± 0.95	-10.32 ± 0.67

Table S2: Binding free energies for the bromosporine selectivity against 22 bromodomains. Values are in kcal/mol.

Protein-ligand system	Experiment	FEP no HREX apo	FEP no HREX holo	FEP HREX apo	FEP HREX holo	Non-eq TI Jarzynski decoupling	Non-eq TI Jarzynski coupling	Non-eq TI BAR
4w52	-5.19 ± 0.16	-3.84 ± 0.54	-4.70 ± 0.30	-4.53 ± 0.05	-5.06 ± 0.06	-5.42 ± 0.12	-5.55 ± 1.72	-4.07 ± 0.34
4w53	-5.52 ± 0.04	-4.77 ± 0.34	-3.74 ± 0.54	-4.82 ± 0.44	-4.78 ± 0.30	-3.54 ± 2.17	-8.85 ± 2.91	-4.98 ± 0.32
4w54	-5.76 ± 0.07	-5.46 ± 0.34	-6.38 ± 0.16	-6.00 ± 0.14	-6.28 ± 0.32	-6.95 ± 0.39	-5.38 ± 2.25	-4.85 ± 0.42
4w55	-6.55 ± 0.02	-6.54 ± 0.24	-6.67 ± 0.34	-6.96 ± 0.06	-6.96 ± 0.05	-7.03 ± 0.53	-5.66 ± 0.66	-6.41 ± 0.46
4w57	-6.70 ± 0.02	-5.81 ± 0.37	-6.47 ± 0.35	-5.89 ± 0.05	-6.34 ± 0.04	-7.95 ± 0.34	-2.90 ± 0.64	-5.42 ± 0.39

Table S3: Binding free energies for the ligands binding to T4 lysozyme. For the FEP and HREX FEP cases, apo and holo denote the states of the starting structures for molecular dynamics simulations: 4w51 was used for apo simulations; for the holo simulations a corresponding holo structure was selected from 4w52, 4w53, 4w54, 4w55 and 4w57. For the non-equilibrium TI calculations, Jarzynski decoupling simulations were initialized with a corresponding holo structure, Jarzynski coupling runs were initialized with the apo structure. Non-equilbrium TI BAR calculations were initialized with the holo and apo structures in the ligand decoupling and coupling directions, respectively. Values are in kcal/mol.

Supplementary References

[1] Hahn, A. M.; Then, H., Measuring the convergence of Monte Carlo free-energy calculations. *Physical Review E* **2010,** *81* (4), 041117.
