# Supplementary material for: Accurate absolute free energies for ligand–protein binding based on non-equilibrium approaches
Source: Commun Chem. 2021 May 11;4:61. doi: 10.1038/s42004-021-00498-y (PMC9814727; doi:10.1038/s42004-021-00498-y)
Supplement: Supplementary file 2 — Description of Additional Supplementary Files [file 42004_2021_498_MOESM2_ESM.pdf]

Description of Additional Supplementary Files

File Name: Supplementary Data 1

Description: Free energy values reported in the Figures 2-8
